# Technical-efficiency analysis of end-of-life care in long-term care facilities within Europe: A cross-sectional study of deceased residents in 6 EU countries (PACE)

**DOI:** 10.1371/journal.pone.0204120

**Published:** 2018-09-25

**Authors:** Anne B. Wichmann, Eddy M. M. Adang, Kris C. P. Vissers, Katarzyna Szczerbińska, Marika Kylänen, Sheila Payne, Giovanni Gambassi, Bregje D. Onwuteaka-Philipsen, Tinne Smets, Lieve Van den Block, Luc Deliens, Myrra J. F. J. Vernooij-Dassen, Yvonne Engels, on behalf of the PACE consortium

**Affiliations:** 1 Radboud University Medical Center, Radboud Institute for Health Sciences, IQ healthcare, Nijmegen, The Netherlands; 2 Radboud University Medical Center, Department for Health Evidence, Nijmegen, The Netherlands; 3 Radboud University Medical Center, Department of Anesthesiology, Pain and Palliative Medicine, Nijmegen, The Netherlands; 4 Unit for Research on Aging Society, Epidemiology and Preventive Medicine Chair, Jagiellonian University Medical College, Kraków, Poland; 5 National Institute for Health and Welfare, Helsinki, Finland; 6 Division of Health Research, Lancaster University, Lancaster, England; 7 Faculty of Medicine and Surgery, Università Cattolica del Sacro Cuore, Rome, Italy; 8 VUmc, Department of Public and Occupational Health, Amsterdam Public Health Research Institute, Amsterdam, The Netherlands; 9 End-of-Life Care Research Group, Vrije Universiteit Brussel & Ghent University, Brussels, Belgium; University of Alberta, CANADA

## Abstract

**Background:**

An ageing population in the EU leads to a higher need of long-term institutional care at the end of life. At the same time, healthcare costs rise while resources remain limited. Consequently, an urgency to extend our knowledge on factors affecting efficiency of long-term care facilities (LTCFs) arises. This study aims to investigate and explain variation in technical efficiency of end-of-life care within and between LTCFs of six EU countries: Belgium (Flanders), England, Finland, Italy, the Netherlands and Poland. In this study, technical efficiency reflects the LTCFs’ ability to obtain maximal quality of life (QoL) and quality of dying (QoD) for residents from a given set of resource inputs (personnel and capacity).

**Methods:**

Cross-sectional data were collected by means of questionnaires on deceased residents identified by LTCFs over a three-month period. An output-oriented data-envelopment analysis (DEA) was performed, producing efficiency scores, incorporating personnel and capacity as input and QoL and QoD as output. Scenario analysis was conducted. Regression analysis was performed on explanatory (country, LTCF type, ownership, availability of palliative care and opioids) and case mix (disease severity) variables.

**Results:**

133 LTCFs of only one type (onsite nurses and offsite GPs) were considered in order to reduce heterogeneity. Variation in LTCF efficiency was found across as well as within countries. This variation was not explained by country, ownership, availability of palliative care or opioids. However, in the *‘hands-on care at the bedside’* scenario, i.e. only taking into account nursing and care assistants as input, Poland (p = 0.00) and Finland (p = 0.04) seemed to be most efficient.

**Conclusions:**

Efficiency of LTCFs differed extensively across as well as within countries, indicating room for considerable efficiency improvement. Our findings should be interpreted cautiously, as comprehensive comparative EU-wide research is challenging as it is influenced by many factors.

## Introduction

Good-quality care is needed for the ageing populations in the European Union (EU).[1, 2] While many countries aim to enable people to continue living in their own home, need of care in long-term care facilities (LTCFs) is growing.[3] This trend is costly for health care systems, and demands the assessment of efficiency of these facilities. Therefore, this study provides insight into variability in efficiency of LTCFs within and between six EU countries (Belgium–Flanders only, England, Finland, Italy, the Netherlands and Poland).

The ageing of our societies is substantially changing healthcare needs [4] and end-of-life (EoL) issues, and gives rise to concerns about quality of life (QoL) and quality of dying (QoD). Since a significant proportion of people live and eventually die in LTCFs, [5, 6], enabling people to live and die well grows in importance.[3] At the same time, long-term care delivery will continue driving up public spending,[7] as LTCFs in the EU are mostly funded out of public money.[7] Illustrating the cost issue: the proportion of long-term care costs in total healthcare expenditure currently are around 40% in the Netherlands and in Finland.[8] A recent FP7 European project (ANCIEN) has shown that a significant share of public expenditures is devoted to institutional care.[9] This trend is a major concern for most governments,[7] and the financing of EoL care in LTCFs will be up for discussion as these issues increasingly affect more people. The World Health Report 2000 called attention to the importance of efficiency in health systems and in achieving the goals of health improvement, responsiveness and fairness in financing.[10] The (balance between) quality of care and efficiency in LTCFs is a growing concern in many industrialized countries.

Thus, it is crucial to internationally compare the efficiency of LTCFs in different EU countries, and to identify factors affecting efficiency and quality of care. Yet, only few single country analyses have been performed in the EU,[11, 12] none of which specifically concerning the EoL. Björkgren et al. found that larger units were seemingly more efficient than smaller ones.[11] Garavaglia and colleagues indicated that private nursing homes appear to outperform public ones.[12] Other studies from Canada and the U.S. primarily focused on ownership as efficiency predictor. Lee et al., for example, found that for-profit facilities were more efficient than not-for-profit ones.[13]

The aim of our study was to investigate and explain variation in the technical efficiency of EoL care within and between LTCFs throughout six EU countries participating in the EU funded 7^th^ Framework Program ‘PACE’ (Palliative Care for Older People in Care and Nursing Homes in Europe).[3] Technical efficiency in this study reflects the LTCFs ability to obtain *maximal* output in terms of of QoL and QoD from a given input of personnel and capacity.

## Methods

### Study population and data

In this exploratory study, data were collected within the EU-funded PACE project. In PACE, a cross-sectional study was performed in 322 LTCFs over six EU countries (Belgium–Flanders only, England, Finland, Italy, the Netherlands and Poland), identifying 1707 deceased residents.[3] Participating LTCFs reported all deaths of residents in and outside the facilities over a three-month period, and questionnaires filled in by the LTCF’s key person and a nurse or care assistants most involved in the care were used (S2 File). Ethics approval from the relevant ethics committees were obtained in all participating countries. Belgium: Commissie Medische Ethiek UZBrussel, 27/05/2015; England: NHS–NRES Committee North West-Haydock, 10/09/2015; Finland: Terveyden jahyvinvoinnin laitos, Institutet för hälsa och välfärd, 30/6/2015; Italy: Comitato Etico, Universita Cattolica del Sacro Cuore, 6/11/2017; Netherlands: Medisch Ethische Toetsingscommissie VU Medisch Centrum, 2/7/2015; Poland: Komisja Bioetycza, Uniwersytetu Jagiellonskiego, 25/6/2015.Details on the study protocol have been published earlier.[3]

### Production function

A data envelopment analysis (DEA) as method of technical efficiency analysis was chosen in this study since in this type of analysis, the efficiency of so-called decision making units (DMU’s) can be calculated. Efficiency scores of 1 indicate maximal efficiency. DMU’s with a score of 1 are used as benchmark for other participating LTCFs. Scores above 1 are considered less efficient. For example; an efficiency score of 1.3 indicates that it is possible to ‘produce’ 30% more output with the same inputs. Moreover, DEA can deal with multiple inputs and outputs, and needs no assumptions about the distribution between them.

**Fig 1:**
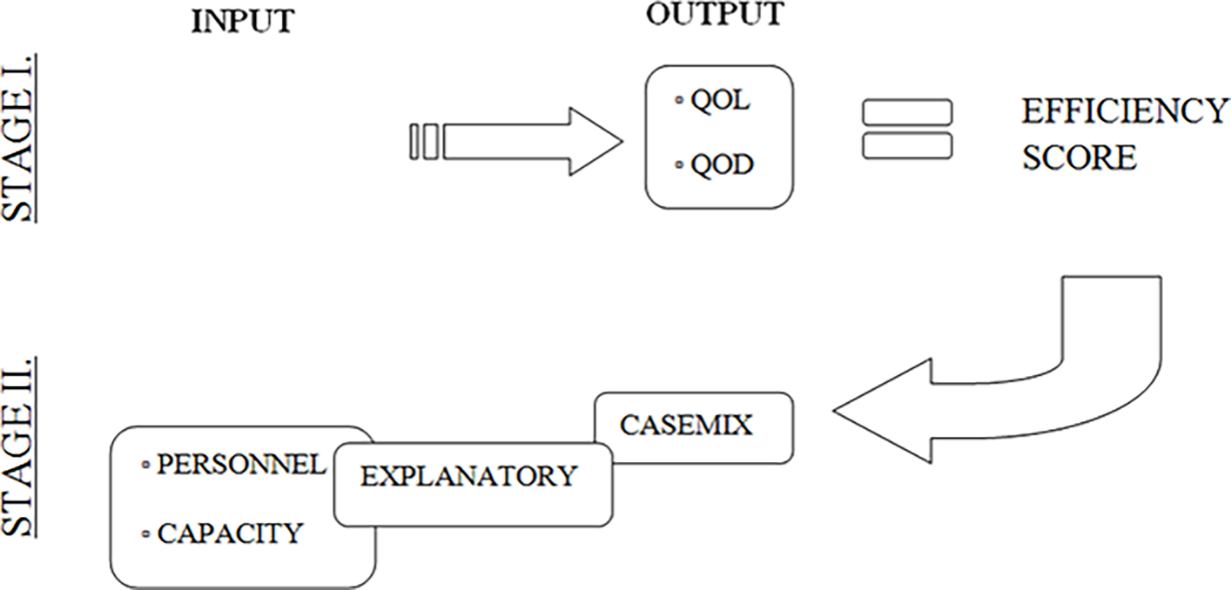
Production function.

In our DEA, LTCFs were handled as DMU’s where output is produced using a given number of resource inputs. The two-stage approach as suggested by Coelli et al. was adopted,[14] in which the first stage analysis involves solving a DEA problem (including only traditional inputs and outputs), and in the second stage efficiency scores from the first stage are regressed upon environmental variables. A quality-driven production function was conducted.[15] See Fig 1 for an illustration of our production function (stage 1), producing efficiency scores, and regression analysis (stage 2) on these scores. The selection of input, output, case mix and explanatory variables (see below) was based on literature as well as the expertise of PACE consortium members.[11, 12]

#### Input variables

Data regarding LTCFs fulltime-equivalents (FTEs) of nurses–both registered and licensed practical, care assistants and allied health professionals (paramedics) were used. These FTEs were divided by the number of beds within LTCFs (indirectly adjusting for case mix), which number subsequently was multiplied by the occupancy rate. In this way *capacity* was also taken into account. GP visits were used as a proxy for the GPs involvement (since FTEs for GPs were not available in all countries as in most LTCFs they are not part of the permanent staff). Our approach was similar to that of Björkgren et al. and Garavaglia et al. by taking into account health, nursing and accommodation (capacity) as resource input.[11, 12]

#### Output variables

Since in LTCFs, (maintaining) QoL and QoD are the most important outcomes, these were chosen as output variables. With regard to QoL, the EuroQol-5D (EQ5D) was used since this is the most often used outcome measure in economic evaluations in health care. The EQ5D is a standardized instrument measuring five health domains (mobility, self-care, usual activities, pain/discomfort and anxiety/depression).[16] In order to achieve utility scores–ranging from 0 (death) to 1 (perfect health)–for the EQ5D, value sets from England were used,[17] since value sets were not yet available for all participating countries.[18] However, the EQ5D arguably is not the most relevant tool for measuring QoL in EoL care, since it takes into account health related domains which are considered less relevant than other dimensions of QoL such as peace, emotions, spiritual and psychosocial well-being.[19] Therefore, two QoD outcome measures were additionally used as output variables. The Comfort Assessment in Dying with Dementia (EOLD-CAD), with four subscales: physical distress, dying symptoms, emotional distress, and well-being,[20] and the Quality of Dying in Long-Term Care (QOD-LTC), comprising of three subscales: personhood, closure, and preparatory tasks.[21] In both tools, higher mean scores reflect a higher quality of end-of-life. Both tools were rated by the nurse or care assistant most closely involved in care of the residents. Literature was consulted and potential output variables were discussed with experts of the PACE consortium. See Table 1 for all input and output variables, and their level of measurement.

**Table 1:** Input and output variables.

INPUT (X)		OUTPUT (Y)	
X1	FTE. NURSING (REGISTRED AND LICENSED PRACTICAL) / TOTAL BEDS / OCC. RATE *–measured on facility level*	Y1	COMFORT ASSESSMENT OF DYING (EOLD-CAD) *–measured on resident level (last week)*
X2	FTE. CARE ASSISTANTS / TOTAL BEDS / OCC. RATE–*measured on facility level*	Y2	QUALITY OF DYING (QOD-LTC) score *–measured on resident level (last month)*
X3	FTE. ALLIED HEALTH PROFESSIONALS (PARAMEDICS) / TOTAL BEDS / OCC. RATE *–measured on facility level*	Y3	EUROQOL 5D5L (EQ5D-5L) utilities *–measured on resident level (last week)*
X4	NUMBER OF GP VISITS *–measured on resident level (last month)*

### Data analysis

In order to ensure representativeness of the data used, descriptive statistics were produced on the data before (total set) and after (subset) deleting missing data to arrive a complete case scenario. Since analyses were done on LTCF level, data were aggregated accordingly.

### Stage 1. Output-oriented data envelopment analysis (DEA)

In stage 1 of the analysis an output-oriented or ‘quality-driven’ DEA, generating efficiency scores, was performed in order to determine the relative efficiency of participating LTCFs. As a result, a ‘frontier’ of most efficient LTCFs was achieved. The distance of individual LTCF efficiency scores from this efficient frontier reflected their relative (in)efficiency. To illustrate this: efficiency scores of 1 indicate a position on the technical efficient frontier. LTCFs with a score of 1 are used as benchmark for other participating LTCFs. Scores above 1 are considered less efficient. For example; an efficiency score of 1.3 indicates it is possible to ‘produce’ 30% more output with the same amount of inputs. Furthermore, an output-oriented constant return to scale approach was adopted: technical efficiency in this study therefore reflects the ability of a LTCF to obtain *maximal* output in QoL and QoD from a given set of resource input of personnel and capacity.

The difference between the efficiency scores and the efficient frontier reflects the relative (in)efficiency. However, since a non-parametric DEA does not take into account sampling variation (statistical noise and random error always underlie samples) this was simulated by means of the bootstrap replications method of Simar and Wilson,[22] a method allowing the assignment of measures of accuracy to sample estimates.[12, 23]

### Stage 2. Truncated regression analysis

In order to explain differences in (bias corrected) efficiency scores from stage 1, truncated regression analysis was performed on a preselected set of explanatory and case mix adjusting variables. Belgium (Flanders) was used as reference country in the regression analysis.

#### Explanatory and case mix variables

Country, status (ownership) and availability of palliative care and opioids were chosen as explanatory variables (see Table 2). Moreover, as technical efficiency differences of LTCFs were conditional on case mix, we made the comparison more homogeneous by taking into account the seven-item Bedford Alzheimer nursing-severity scale score (BANS-S, a rating scale (range 7–28) comprising cognitive and functional items) of deceased residents, percentage of residents needing assistance in eating, and the mean length of stay of residents in the LTCFs were used (see Table 3).

**Table 2:** Explanatory variables.

VARIABLE	EXPLANATION
Country	1 = Belgium (Flanders) 2 = Finland 3 = Italy 4 = Netherlands (NL) 5 = Poland 6 = United Kingdom (ENGLAND)
Status	1 = public-nonprofit 2 = private-nonprofit 3 = private-profit
Palliative care	1 = palliative care team OR advice available 2 = palliative care team OR advice not available
Opioids	1 = opioids available 24/7 2 = opioids not available 24/7

**Table 3:** Case mix variables.

VARIABLE	EXPLANATION
Bedford Alzheimer Nursing Severity Scale (BANS-S)	Scale measuring disease severity (*resident level*)
Number of residents needing assistance with eating	Number of residents needing assistance in eating (*LTCF level*)
Average length of stay in facility	Average length of stay in facility (*LTCF level*)

### Base case and scenario analysis

Type 2 LTCFs (with onsite nurses and offsite GPs) were most abundant in the dataset (N = 135), and this was the only type that was present in every country (Table g in S1 Tables). Since DEA can best deal with homogeneous data when calculating efficiency scores, and this was not the case when comparing all types of LTCFs (Tables e-f in S1 Tables), all analyses were conducted on type 2 LTCFs only. See Table 2 for the explanation of LTCF types.

Next to the *base case* analysis, taking into account all input variables, a *scenario analysis* was performed. Since hands-on care at the bedside (FTEs nursing and care assistants) deliver the large majority of care in LTCFs, [24, 25] they are particularly important in producing QoL and QoD in LTCFs. Therefore, another scenario analysis only taking into account this *hands-on care at the bedside* was conducted.

## Results

Of the 322 participating LTCFs, 176 (54,6%) had complete cases on all in- and output variables. After deleting LTCFs with missing values on explanatory and case mix variables, 173 LTCFs (53,7%) remained. PACE questionnaires regarding facility characteristics were completed for 94,7% LTCFs, response rates for questionnaires regarding the resident filled in by the staff ranged from 95,1% in Finland to 54,2% in England.

### Descriptive statistics & representativeness

#### Descriptive statistics on input, output and regression variables before (total set) and after (analysis set) deleting missing data for the complete set of LTCFs can be found in S1 Tables.

**Input variables**

Comparing the input variables in the total versus the analysis set showed no considerable differences (Tables a-b in S1 Tables). England had high (0,7), and Finland–and Poland, to a lesser extent–rather low (0,07 and 0,17) FTEs care assistants per bed. With regard to FTEs nursing (including both registered and licensed practical nurses), both the Netherlands (0,07) and England (0,09) had quite low, and Finland (0,56) rather high numbers per bed. Moreover, FTEs allied health professionals (paramedics) were lowest in England (0,00), Finland (0,03) and Belgium (0,04), and relatively high in Poland (0,12), Italy (0,10) and the Netherlands (0,09). GP visits per resident in the last month of life were highest in the Netherlands (7,6) and Italy (6,7) and lowest in Finland (3,3) and England (3,5).

We found that, in the countries overall, type 1 and 2 LTCFs had about equal FTEs nursing (0,21 and 0,31 respectively) and care assistants (ca. 0,25) per bed, and relatively more GP visits per resident in the last month of life (6,8 and 4,7 respectively). As expected, type 3 LTCFs had virtually no FTEs nursing and allied health professionals (paramedics), but many care assistants (0,76).

**Output variables**

Output variables before and after deleting missing data were also comparable (Tables c-d in S1 Tables). Data on QoL and QoD measures showed that outputs for QoL and QoD were highest in England (EOLD-CAD: 33,5, QOD-LTC: 43,3, EQ5D: 0,27) and in type 3 LTCFs (EOLD-CAD: 34,4, QOD-LTC: 43,6, EQ5D: 0,28), which were only present in England. QoL as measured by the EQ5D was highest in England and Belgium (0,27 and 0,26 respectively), and by far lowest in the Italian LTCFs (0,08), followed by Polish (0,17) and Finnish (0,18) LTCFs. QoD as measured by the EOLD-CAD and QOD-LTC was highest in England (33,5 and 43,3). QoD as measured by the EOLD-CAD was lowest in Belgium (30,1) and Poland (30,1). QoD as measured by the QOD-LTC was lowest in the Netherlands (37,4) and Finland (37,8).

**Regression: case mix variables**

Regarding case mix (disease severity), the BANS-S score of deceased residents, percentages of all residents in the LTCFs needing assistance in eating and the mean length of stay of all residents in the LTCFs are described. As can be seen in Tables e-f (in S1 Tables), means of case mix variables were comparable in total and analysis sets. Disease severity was relatively low in LTCFs throughout England, the Netherlands and Belgium (BANS-S 16,9, 17,4 and 18,0 respectively), and lowest in type 3 LTCFs (BANS-S 17,1), which were only present in England.

**Regression: explanatory variables**

Only type 2 LTCFs were included in all countries (Table g in S1 Tables). Italy, the Netherlands and Poland included type 1 (onsite day and night care from physicians and nurses) and 2 (onsite care from nurses and care from GPs based offsite) LTCFs. Belgium and Finland only included type 2. England included type 2 and 3 (offsite care from nurses and GPs) LTCFs. With regard to ownership; the Netherlands only included public, and England only private (profit and non-profit) LTCFs. All other countries included LTCFs with different ownership structures. Distributions stayed the same before and after deleting missing data (Table h in S1 Tables). Availability of palliative care and opioids was highest in Belgium (92,9%) and lowest in Italy (48,3%) and Poland (51%) (Table i in S1 Tables).

### Inferential statistics

#### Of the 305 LTCFs, 176 (58%) could be taken into account in the stage 1 analysis. Because of heterogeneity in disease severity and QoL and QoD of residents per LTCF type (1, 2 and 3), in the base case analyses another 41 LTCFs were excluded because of not being type 2. Eventually, 135 LTCFs (34%) were taken into account in the *base case* analysis

**Stage 1. Output-oriented DEA**

The stage 1 DEA showed that 48 out of 135 LTCFs (35%) were on the efficient frontier, meaning these LTCFs served as benchmarks for the other LTCFs. The results point to a significant difference from the efficient frontier (p < 0.01, mean efficiency score 1.56, 95%-CI: 1.45–1.66), indicating that–based on our production function–there is room for efficiency improvement. Next to this variation across all LTCFs, a significant difference in efficiency was found *within* countries (Table 4).

**Table 4:** Bias-corrected efficiency scores per country (the closer to ‘1’, the more efficient).

Country	Mean	Std. Dev.	P-value
Belgium	1.61	0.44	<0.01
Finland	1.49	0.52	<0.01
Italy	1.98	1.01	<0.01
NL	1.36	0.44	0.02
Poland	1.21	0.37	0.02
England	1.03	0.08	0.15

**STAGE 2. Truncated regression analysis–base case**

After having obtained the bias-corrected efficiency scores in stage 1, truncated regression analysis was performed in order to explain differences in the scores. Due to missing values on explanatory variables, 133 LTCFs (out of the 135 in stage 1 remaining) were taken into account in the stage 2 analysis.

The regression analysis indicated considerable variability in LTCF efficiency throughout Europe (Table 5). The variation between countries however, did not differ significantly from benchmark country Belgium. Similarly, ownership, availability and/or use of palliative care or opioids did not influence efficiency.

**Table 5:** Regression analysis (*base case–all input*, *type 2 LTCFs*).

	Coef. (β)	P-value	95%—CI
**Country** *(reference*: *Belgium)*			
Finland	-1.53	0.38	-5.0–1.9
Italy	1.39	0.40	-1.9–4.7
NL	-0.16	0.93	-4.0–3.7
Poland	0.03	0.99	-4.4–4.4
England	-13.31	0.11	-29.8–3.1
**Status** *(reference*: *public)*			
Private-nonprofit	1.40	0.28	-1.1–3.9
Private-profit	1.73	0.33	-1.8–5.2
**Palliative care** (team / advice) *(reference*: *no)*			
Yes	-1.08	0.44	-3.8–1.7
**Opioids** (availability) *(reference*: *no)*			
Yes	0.79	0.68	-2.9–4.5
**BANS-S**	-0.13	0.58	-0.6–0.3
**% assistance eating**	-0.55	0.87	-7.1–6.0
**Length of stay**	-0.00	0.14	-0.0–0.0

#### Scenario analysis

When conducting the *hands-on care at the bedside* analysis (for type 2 LTCFs), Poland (p = 0.00) and Finland (p = 0.04) seemed to be more efficient than the benchmark Belgium. Again, ownership, availability and/or use of palliative care or opioids did not influence efficiency (Table 6).

**Table 6:** Regression analysis (*Hands-on care–*nursing and care–at the bedside, type 2 LTCFs).

	Coef. (β)	P-value	95%—CI
**Country** *(reference*: *Belgium)*			
Finland	-1.86	**0.04**	-2.9 –-0.1
Italy	0.15	0.86	-0.4–2.1
NL	-1.05	0.18	-2.3–0.4
Poland	-3.87	**0.00**	-6.0 –-1.8
England	-0.38	0.73	-1.2–2.2
**Status** *(reference*: *public)*			
Private-nonprofit	-0.55	0.35	-1.2–0.6
Private-profit	0.67	0.40	-0.7–1.9
**Palliative care** (team / advice) *(reference*: *no)*			
Yes	-0.18	0.74	-1.8–1.2
**Opioids** (availability) *(reference*: *no)*			
Yes	-0.51	0.66	-1.6–1.5
**BANS-S**	0.15	0.59	-0.1–0.2
**% assistance eating**	1.02	0.42	-1.5–3.5
**Length of stay**	-0.00	0.88	-0.0–0.0

## Discussion

In this study, a quality-driven production function consisting of personnel (nurses, care assistants, allied health professionals/paramedics and GPs) and capacity (bed occupancy) as input, and QoL (EQ5D) and QoD (EOLD-CAD and QOD-LTC)–as judged by staff—as output was built to calculate technical efficiency scores for each LTCF. Country, type, ownership and the availability of palliative care and opioids were pre-selected to explain variability in efficiency. A *base case* analysis and a *scenario* analysis were conducted. A lot of variation in technical efficiency of partaking LTCFs was found, which persisted when looking within countries.

Both a *base case* analysis as well as a *hands-on care at the bedside* scenario were conducted. The latter as nurses and care assistant deliver the large majority of care in LTCFs,[24, 25] and they thus particularly are important in producing QoL and QoD. In order to improve heterogeneity, only type 2 LTCFs–with onsite care from nurses and care from offsite based GPs–were taken into account in our analyses. Efficiency scores suggest the English LTCFs tend to be more efficient, but this difference was not statistically significant. Looking at the included LTCFs from England in more detail, we observed they have a distinctive model characterized by relatively few nurses, many care assistants, and an emphasis on palliative care). Variation in efficiency did significantly differ between countries in the *hands-on care at the bedside* scenario, in which Poland and Finland seemed most efficient. In Poland, relatively good QoL and QoD was achieved despite low–possibly even understaffed–resource input. Moreover, it was noteworthy that in Finland the ‘hands-on care at the bedside’ is relatively high educated. I.e. the ratio nurses (registered and licensed practical) over care assistants is higher than in other participating countries. In this study, however, we only studied whether the LTCFs’ country is influencing efficiency, not why certain countries are more efficient. Future analyses of contextual frameworks would be important to explain further differences in efficiency.

Only resident-related data concerning the last weeks of life was used, making the group very homogeneous in terms of phase of life. However, variation in disease severity and closely related LTCF types was found, which can hardly be standardized. This was a disadvantage, since comparative research, especially DEA, is best undertaken with homogeneous data. Moreover, output (QoL & QoD) and disease severity (BANS-S) data were gathered among *deceased* residents, while at the same time input data (FTE) was based on the entire LTCF. Ideally, data of *all* LTCF residents would have been considered. Also as this gives insight in the comparability of LTCFs as a whole. This is challenging however, as it requires prospective data collection.

Moreover, healthcare professionals not having an appointment in the facility, who visit as required, were missed in our analyses although they and family or volunteers might also influence QoL and QoD of residents. Another limitation regards the fact that questionnaires regarding the deceased resident were filled in by a care provider from the LTCF staff. The knowledge level and understanding of questions–i.a. regarding the residents’ QoL and QoD–by the care provider might have influenced their answers; the use of outcomes judged by the staff might have led to an overestimation of QoL and QoD in countries where education in palliative care in LTCFs is low. Moreover, value sets from England were used to derive EQ5D utilities, as they are not yet available for all participating countries.[17] It would be valuable for future studies to be able using population-specific valuation sets better reflecting cultural differences.[26]

This pioneering large-scale study provides a first insight into the technical efficiency of end-of-life care delivery in LTCFs in six European countries. In terms of methodology our study is comparable to others.[11, 12] However, it is the first to make a comparison specifically in EoL care and across countries. Also, the fact that we did not follow the standard choice of using quantity (volumes) as output, but used quality is a strength.[27] Comprehensive comparative EU-wide research remains a challenge as it is influenced by many factors.

## Supporting information

S1 Tables Descriptive statistics on input, output, explanatory and case mix variables. (DOCX)

S1 File PACE questionnaires used. (ZIP)

S1 Dataset PACE dataset base case. (SAV)
