# Correction: Technical-efficiency analysis of end-of-life care in long-term care facilities within Europe: A cross-sectional study of deceased residents in 6 EU countries (PACE)

**DOI:** 10.1371/journal.pone.0208199

**Published:** 2018-11-21

**Authors:** Anne B. Wichmann, Eddy M. M. Adang, Kris C. P. Vissers, Katarzyna Szczerbińska, Marika Kylänen, Sheila Payne, Giovanni Gambassi, Bregje D. Onwuteaka-Philipsen, Tinne Smets, Lieve Van den Block, Luc Deliens, Myrra J. F. J. Vernooij-Dassen, Yvonne Engels, on behalf of the PACE consortium

Fig 1 is incorrect. The authors have provided a corrected version here.

**Fig 1:**
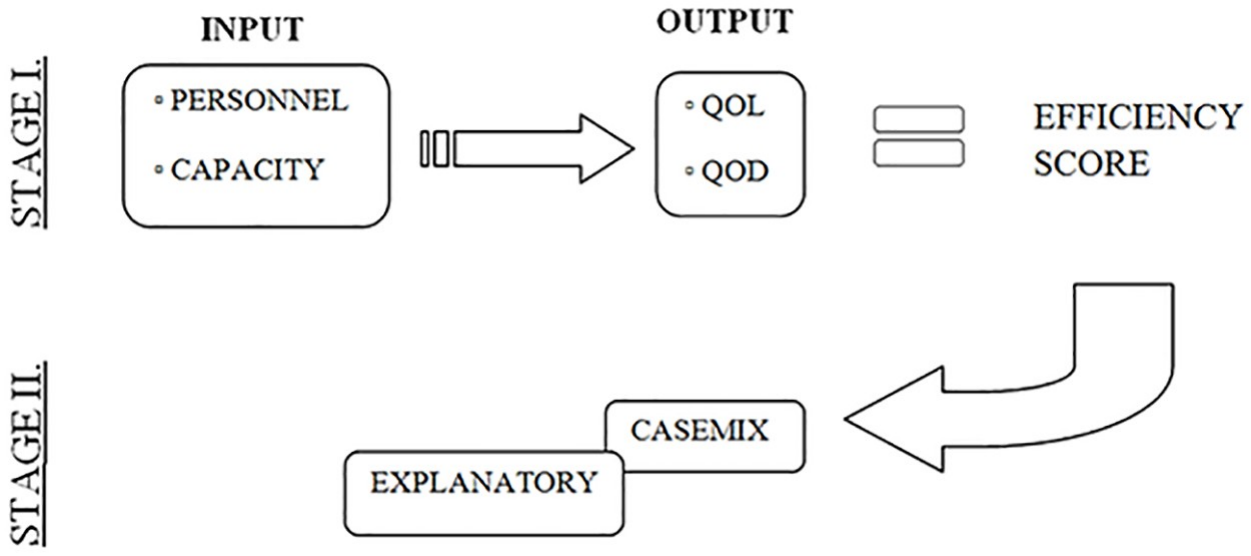
Production function.
