# Supplementary material for: Technical-efficiency analysis of end-of-life care in long-term care facilities within Europe: A cross-sectional study of deceased residents in 6 EU countries (PACE)
Source: PLoS One. 2018 Sep 25;13(9):e0204120. doi: 10.1371/journal.pone.0204120 (PMC6155520; doi:10.1371/journal.pone.0204120)
Supplement: S1 Tables — (DOCX) [file pone.0204120.s001.docx]

**S1 Tables. Descriptive statistics input, output, explanatory and case mix variables.**

S1 Tables Table a. Personnel (GP visits, FTE nursing, FTE care assistants) in last month of life per country – total vs. analysis group (incl. 95%-CI)

|  | **GP visits** | | **FTE nursing** (registered and licensed practical)***** | | **FTE care assistants*** | | **FTE allied health professionals** (paramedics)***** | |
| --- | --- | --- | --- | --- | --- | --- | --- | --- |
|  | *Total* | *Analysis* | *Total* | *Analysis* | *Total* | *Analysis* | *Total* | *Analysis* |
| Belgium | 5,0 *(4,2-5,9)* | 5,0 *(4,1-5,9)* | 0,17  *(0,15-0,19)* | 0,16 *(0,15-0,18)* | 0,24 *(0,22-0,26)* | 0,25 *(0,22-0,27)* | 0,04 *(0,04-0,05)* | 0,05 *(0,04-0,06)* |
| Finland | 3,3 *(2,6-4,0)* | 3,2 *(2,1-4,3)* | 0,56 *(0,53-0,60)* | 0,60 *(0,5-0,6)* | 0,08 *(0,06-0,01)* | 0,09 *(0,06-0,11)* | 0,03 *(0,02-0,04)* | 0,03 *(0,01-0,04)* |
| Italy | 6,7 *(2,7-10,7)* | 7,4 *(3,1-12,4)* | 0,20 *(0,16-0,25)* | 0,19 *(0,13-0,24)* | 0,42 *(0,36-0,47)* | 0,41 *(0,34-0,49)* | 0,10 *(0,06-0,14)* | 0,10 *(0,05-0,12)* |
| NL | 7,6 *(6,2-9,1)* | 8,3 (*5,7-11,0)* | 0,07 *(0,05-0,10)* | 0,09 *(0,05-0,13)* | 0,42 *(0,37-0,47)* | 0,40 *(0,33-0,48)* | 0,09 *(0,00-0,18)* | 0,10 *(-0,0-0,22)* |
| Poland | 5,7 *(4,2-7,2)* | 6,0 *(4,3-7,9)* | 0,16 *(0,13-0,20)* | 0,17 *(0,13-0,22*) | 0,17 *(0,14-0,20)* | 0,16 *(4,1-5,9)* | 0,12 *(0,09-0,15)* | 0,13 *(0,13-0,19)* |
| England | 3,5 *(2,7-4,3)* | 3,6 *(2,5-4,7)* | 0,09 *(0,06-0,12)* | 0,09 *(0,05-0,13)* | 0,72 *(0,62-0,82)* | 0,69 *(0,55-0,83)* | 0,00 *(0,00-0,01)* | 0,00 *(0,00-0,00)* |
| Mean | 5,0 *(4,4-5,6)* | 5,3 *(4,5-6,1)* | 0,27 *(0,25-0,30)* | 0,26 *(0,22-0,29)* | 0,29 *(0,26-0,33)* | 0,28 *(0,24-0,32)* | 0,06 *(0,05-0,08)* | 0,06 *(0,05-0,08)* |

* divided by the total number of beds within LTCFs, multiplied by the occupancy rate

S1 Tables Table b. Personnel (GP visits, FTE nursing, FTE care assistants) in last month of life per LTCF type – total vs. analysis group (incl. 95%-CI)

|  | **GP visits** | | **FTE nursing** (registered and licensed practical)***** | | **FTE care assistants*** | | **FTE allied health professionals** (paramedics)***** | |
| --- | --- | --- | --- | --- | --- | --- | --- | --- |
|  | *Total* | *Analysis* | *Total* | *Analysis* | *Total* | *Analysis* | *Total* | *Analysis* |
| Type 1 | 6,8 *(4,9-8,7)* | 7,3 *(4,9-9,8)* | 0,21 *(0,17-0,25)* | 0,23 *(0,18-0,27)* | 0,25 *(0,19-0,32)* | 0,22 *(0,16-0,28)* | 0,14 *(0,08-0,20)* | 0,13 *(0,07-0,19)* |
| Type 2 | 4,7 *(4,1-5,3)* | 5,0 *(4,1-5,9)* | 0,31 *(0,27-0,34)* | 0,28 *(0,24-0,32)* | 0,26 *(0,23-0,29)* | 0,26 *(0,22-0,29)* | 0,05 *(0,04-0,06)* | 0,05 *(0,04-0,07)* |
| Type 3 | 3,2 *(2,0-4,4)* | 3,2 *(1,5-4,8)* | 0,01 *(0,01-0,03)* | 0,02 *(-0,01-0,05)* | 0,76 *(0,59-0,93)* | 0,78 *(0,49-1,06)* | 0,00 *(0,00-0,00)* | 0,00 *(0,00-0,00)* |

* divided by the total number of beds within LTCFs, multiplied by the occupancy rate

S1 Tables Table c. QoL and QoD per country – total vs. analysis group (incl. 95%-CI)

|  | **EOLD-CAD** | | **QOD-LTC** | | **EQ5D** | |
| --- | --- | --- | --- | --- | --- | --- |
|  | *Total* | *Analysis* | *Total* | *Analysis* | *Total* | *Analysis* |
| Belgium | 30,1 *(29,1-31,1)* | 29,9 *(28,7-31,1)* | 39,5 *(37,9-41,2)* | 39,5 *(37,5-41,4)* | 0,26 *(0,21-0,30)* | 0,24 *(0,20-0,28)* |
| Finland | 31,1 *(30,4-31,7)* | 31,3 *(30,3-32,2)* | 37,8 *(36,9-38,8)* | 38,0 *(36,8-39,3)* | 0,18 *(0,16-0,21)* | 0,19 *(0,16-0,22)* |
| Italy | 31,5 *(30,3-32,8)* | 31,1 *(30,0-32,9)* | 38,7 *(36,4-41,1)* | 38,1 *(35,1-41,4)* | 0,08 *(0,02-0,15)* | 0,06 *(-0,02-0,14)* |
| NL | 31,0 *(29,7-32,3)* | 31,5 *(29,4-33,5)* | 37,4 *(35,2-39,6)* | 37,6 *(34,2-41,0)* | 0,22 *(0,17-0,27)* | 0,22 *(0,18-0,27)* |
| Poland | 30,1 *(29,0-31,3)* | 30,5 *(29,3-31,6)* | 40,5 *(38,7-42,3)* | 40,1 *(38,2-42,0)* | 0,17 *(0,14-0,21)* | 0,17 *(0,13-0,21)* |
| England | 33,5 *(32,2-34,7)* | 33,1 *(31,4-34,8)* | 43,3 *(41,3-45,2)* | 44,0 *(41,8-46,3)* | 0,27 *(0,21-0,34)* | 0,29 *(0,19-0,39)* |
| Mean | 31,2 (*30,8-31,6)* | 31,1 *(30,6-31,6)* | 39,6 *(38,9-40,3)* | 39,5 *(38,6-40,4)* | 0,19 *(0,18-0,21)* | 0,20 *(0,17-0,22)* |

S1 Tables Table d. QoL and QoD per LTCF type – total vs. analysis group (including 95%-CI)

|  | **EOLD-CAD** | | **QOD-LTC** | | **EQ5D** | |
| --- | --- | --- | --- | --- | --- | --- |
|  | *Total* | *Analysis* | *Total* | *Analysis* | *Total* | *Analysis* |
| Type 1 | 31,1 *(30,0-32,1)* | 31,4 *(30,2-32,6)* | 38,1 *(36,1-40,1)* | 38,9 (*36,1-41,6)* | 0,14 *(0,11-0,18)* | 0,14 *(0,09-0,18)* |
| Type 2 | 30,8 *(30,4-31,3)* | 30,8 *(30,2-31,4)* | 39,1 *(38,4-39,9)* | 39,2 *(38,3-40,1)* | 0,20 *(0,18-0,22*) | 0,20 *(0,18-0,22)* |
| Type 3 | 34,4 *(32,3-36,5)* | 34,6 *(31,5-37,7)* | 43,6 *(40,8-46,4)* | 45,0 *(41,3-48,6)* | 0,28 *(0,16-0,39)* | 0,30 *(0,09-0,50)* |

S1 Tables Table e. Disease severity per country – total vs. analysis group (incl. 95%-CI)

|  | | **BANS-S** | | **% assistance eating** | | **Length of stay (days)** | |
| --- | --- | --- | --- | --- | --- | --- | --- |
|  | | *Total* | *Analysis* | *Total* | *Analysis* | *Total* | *Analysis* |
|  | Belgium | 18,0 *(17,0-18,9)* | 18,2 *(17,3-19,1)* | 19,4 *(16,3-22,5)* | 18,7 (*15,1-22,3*) | 1038 *(914-1162)* | 1024 (*878-1172)* |
|  | Finland | 20,0 *(19,3-20,6)* | 20,7 (*19,9-21,6)* | 42,0 *(36,6-47,5)* | 48,7 *(40,3-57,2)* | 605 *(498-712*) | 676 *(514-839*) |
|  | Italy | 21,9 *(21,0-22,9)* | 22,2 *(21,3-23,2)* | 49,0 *(41,0-56,9)* | 49,5 *(40,7-58,4*) | 380 (*273-487)* | 435 *(286-583)* |
|  | NL | 17,4 *(16,6-18,3)* | 17,8 *(16,6-19,1*) | 21,5 *(15,9-27,2)* | 21,2 (*12,5-29,8)* | 841 *(718-965)* | 856 (*667-1044)* |
|  | Poland | 21,6 *(20,8-22,3)* | 21,6 *((20,8-22,4)* | 37,0 (*30,0-44,0)* | 39,9 (*32,0-47,9)* | 1256 (*885-1626*) | 1187 (*774-1599)* |
|  | England | 16,9 *(15,7-18,1)* | 16,7 (*15,3-18,1*) | 23,2 *(16,3-22,5)* | 23,8 *(14,9-32,9)* | 709 *(543-874)* | 814 *(559-1067)* |
|  | Mean | 19,4 *(19,1-19,8)* | 19,8 *(19,3-20,3)* | 32,9 *(16,3-22,5)* | 35,6 (*31,8-39,3)* | 808 *(721-894)* | 869 *(751-988)* |

S1 Tables Table f. Disease severity per LTCF type - total vs. analysis group (incl. 95%-CI)

|  | | **BANS-S** | | **% assistance eating** | | **Length of stay (days)** | |
| --- | --- | --- | --- | --- | --- | --- | --- |
|  | | *Total* | *Analysis* | *Total* | *Analysis* | *Total* | *Analysis* |
|  | Type 1 | 21,4 *(20,6-22,2)* | 21,8 *(20,9-22,8)* | 44,6 *(36,9-52,2)* | 48,9 *(40,0-58,0)* | 433 *(328-538*) | 326 *(230-423)* |
|  | Type 2 | 19,2 *(18,7-19,6)* | 19,5 *(19,0-20,1)* | 32,4 *(29,4-35,4)* | 34,2 (*30,1-38,4)* | 879 *(775-984)* | 987 *(846-1127*) |
|  | Type 3 | 17,1 *(15,2-19,0)* | 17,1 *(14,7-19,6)* | 15,8 *(8,0-23,7)* | 11,3 *(4,1-18,6)* | 859 *(578-1141*) | 1059 *(524-1594)* |

S1 Tables Table g. Number and type of LTCFs per country – total vs. analysis group

| Country | LTCF type | | | | | | Total | |
| --- | --- | --- | --- | --- | --- | --- | --- | --- |
|  | **Type 1** | | **Type 2** | | **Type 3** | |  | |
|  | Total | Analysis | Total | Analysis | Total | Analysis | Total | Analysis |
| Belgium | 0 | 0 | 43 | 33 | 0 | 0 | 43 | 33 |
| Finland | 0 | 0 | 88 | 43 | 0 | 0 | 88 | 43 |
| Italy | 8 | 4 | 25 | 16 | 0 | 0 | 33 | 20 |
| NL | 17 | 7 | 27 | 11 | 0 | 0 | 44 | 18 |
| Poland | 22 | 20 | 27 | 20 | 0 | 0 | 49 | 40 |
| England | 0 | 0 | 22 | 12 | 26 | 10 | 48 | 22 |
| Total | 47 | 31 | 232 | 135 | 26 | 10 | **305** | **176** |

S1 Tables Table h. Ownership LTCFs per country – LTCF level – total vs. analysis group

| Country | Status | | | | | |
| --- | --- | --- | --- | --- | --- | --- |
|  | **% public (non-profit)** | | **% private non-profit** | | **% private profit** | |
|  | *Total* | *Analysis* | *Total* | *Analysis* | *Total* | *Analysis* |
| Belgium | 44,8 | 42,4 | 44,2 | 48,5 | 7,0 | 9,1 |
| Finland | 75,9 | 88,4 | 11,5 | 7,0 | 12,6 | 4,7 |
| Italy | 31,0 | 35 | 20,7 | 20 | 48,3 | 45 |
| NL | 100 | 100 | 0 | 0 | 0 | 0 |
| Poland | 62,5 | 62,5 | 35,4 | 35 | 2,1 | 2,5 |
| England | 2,5 | 0 | 12,5 | 18,2 | 85 | 81,8 |
| Total | 58,5 | 58 | 19,7 | 23,3 | 21,8 | 18,8 |

S1 Tables Table i. Availability of palliative care (team or advice) and opioids – LTCF level – total vs. analysis group

|  | | **% palliative care** | | **% opioid use** | |
| --- | --- | --- | --- | --- | --- |
|  | | *Total* | *Analysis* | *Total* | *Analysis* |
|  | Belgium | 92,9 | 93,8 | 97,6 | 96,9 |
|  | Finland | 39,8 | 34,9 | 95,4 | 95,3 |
|  | Italy | 48,3 | 45 | 89,7 | 90 |
|  | NL | 69 | 61,1 | 90,5 | 94,4 |
|  | Poland | 51 | 50 | 72,9 | 69,2 |
|  | England | 87,2 | 95,2 | 97,2 | 100 |
|  | Total | 60,9 | 60,3 | 90,9 | 89,6 |
