# Supplementary material for: Technical-efficiency analysis of end-of-life care in long-term care facilities within Europe: A cross-sectional study of deceased residents in 6 EU countries (PACE)
Source: PLoS One. 2018 Sep 25;13(9):e0204120. doi: 10.1371/journal.pone.0204120 (PMC6155520; doi:10.1371/journal.pone.0204120)
Supplement: S1 File — (ZIP) [file pone.0204120.s002.zip › PACE_questionnaire_KPfacility_study1.doc]

**Long term care facility - KEY PERSON FROM MANAGEMENT OR ADMINISTRATION**

**Questionnaire to be completed by long term care facility key person regarding the facility**

*Information regarding facility (fill in only once)*

| **1** | [**Country-specific question]** Status of provider | | - public-nonprofit - private-nonprofit - private-profit | | | | | | |
| --- | --- | --- | --- | --- | --- | --- | --- | --- | --- |
| **2** | [**Country-specific question(s) regarding casemix]**  How many approved beds does your facility have? (*Excluding short stays* ) | | - ROB - RVT | | | | | | |
| **3** | [**Country-specific question(s) regarding casemix]**  Please estimate the number of residents per dependency category based on the most recently complete KATZ-scale: | | Category A (number of residents)  Category B (number of residents)  Category C (number of residents)  Category Cd (number of residents) | | | | | | |
| **4** | [**Country-specific question regarding religious affiliation of facility**] With which umbrella organization is your facility associated? | | - Zorgnet - Ferubel - VLOZO (FOS) - VVSG - other, please specify: - none | | | | | | |
| **5** | Total number of available beds in the facility at this moment: | | | | beds | | | | |
| **6** | Total number of occupied beds in the facility at this moment: | | | | beds | | | | |
| **7** | Number of residents at this moment requiring full assistance in eating:  *(if necessary, make an estimation)* | | | | residents | | | | |
| **8** | Averaged length of stay (days) in the facility:  *(if possible, please base on annual reports, otherwise please make an estimation)* | | | | days | | | | |
| **9** | Level of personnel: [**Country-specific answer categories**]  *(please fill in the Full Time Equivalent (FTE) for each of the different types of personnel currently employed in your facility)* | | **Number of FTE** | | | **Type of personnel** | | | |
|  |  |  |  | | | General practitioner | | | |
|  |  |  |  | | | Registered nurses | | | |
|  |  |  |  | | | Licensed practical nurses | | | |
|  |  |  |  | | | Nursing Aides | | | |
|  |  |  |  | | | Physiotherapists | | | |
|  |  |  |  | | | Psychologists | | | |
|  |  |  |  | | | Social workers | | | |
|  |  |  |  | | | Administrative staff | | | |
|  |  |  |  | | | Other, please specify: | | | |
| **10** | How many volunteers are currently registered in your facility? | | (number of volunteers) | | | | | | |
| **11** | [**Country-specific facility characteristics]**  BE: Is the CRA the general practitioner for all residents? | | | | | | | | - yes |
|  |  |  |  |  |  |  |  |  | - no |
| **12** | [**Country-specific facility characteristics]**  BE: How many hours per week (approximately), is the CRA present in your facility? | | | | | | | | hours per week |
| **13** | How many residents at your facility have died in the past 12 months?  *(please indicate exactly, counting from today)* | | | | | | | | residents |
| **14** | Is there a separate ward for residents with dementia in this facility? | | | | | | | | - yes |
|  |  |  |  |  |  |  |  |  | - no |
| **15** | Does the facility have specific written guidelines with regard to providing palliative care? | | | | | | | | - yes - no - I don’t know |
| **16** | Does the facility use InterRAI?  [**Country-specific question:** other care planning tools can be added to this question in each country, e.g. Gold Standards Framework, Six Steps to Succes**]** | | | | | | | | - yes - no |
| **17** | Are generally applicable patient-specific forms/records available at your facility about prior care planning with regard to the residents’ end of life?  **[Country-specific examples]** BE: *For example “restriction form”, “therapy restriction code”, “agreements form”, “comfort record”, “palliative record”* | | | | | | | | - no - yes, specifically: |
| **18** | ***Specialist palliative care*** *is provided by specialised services for patients with complex problems not adequately covered by other treatment options. Specialist palliative care services require a team approach, combining a multiprofessional team with an interdisciplinary mode of work. Team members must be highly qualified and should have their main focus of work in palliative care.* | | | | | | | | |
|  | Is there a **specialist palliative care team** present in your facility (employed in your facility)? | | | - yes - no 🡪 **continue to question 19** | | | | | |
|  | a. If yes, do you use this specialist palliative care team? | | | - yes, for all residents in need of specialised palliative care - yes, for most residents in need of specialised palliative care - yes, for some residents in need of specialised palliative care - no, never | | | | | |
| **19** | Is specialist palliative care advice available to professionals delivering palliative care in your facility? | | | - yes - no 🡪 **continue to question 20** | | | | | |
|  | a. If yes, do you use this advice? | | | - yes, for all residents in need of specialised palliative care - yes, for most residents in need of specialised palliative care - yes, for some residents in need of specialised palliative care - no, never | | | | | |
| **20** | Are opioids available 24/7 for residents in need of palliative care in your facility? | | | | | | | - yes, for all residents - yes, for most residents - yes, for some residents - no, never | |
| **21** | Does your facility offer residents in need of palliative care an assigned contact person (e.g. care manager, case manager or key worker) who maintains regular contact with the resident and his or her relatives, in order to ensure coordinated delivery of health and social care? | | | | | | | - yes, for all residents - yes, for most residents - yes, for some residents - no, never | |
| **22** | What specialised equipment is available for residents in need of palliative care in your facility? *(multiple answers possible)* | | | | | | | - anti-decubitus mattresses - suction equipment - stoma care supplies - oxygen delivery - drug administration pumps - hospital beds - other, please specify: | |
| **23** | Are single bedrooms available in your facility for ALL residents who are dying and who wish to have one? | | | | | | | - yes - no | |
| **24** | Are there unrestricted visiting hours for relatives of residents who are dying, if they wish? | | | | | | | - yes - no | |
| **25** | Are there facilities for relatives to stay overnight with their dying relative? | | | | | | | - yes - no | |
| **26** | Do you have a procedure in place to ensure that relatives of residents are offered bereavement support, if they need or wish to have support? | | | | | | - yes - no | | |
| **27** | Is there a regular multidisciplinary meeting (with at least a physician and a nurse) to review treatment and care plans organised in your facility? | | | | | | | - yes - no 🡪 **continue to question 28** | |
|  | a. If yes, how frequently is this meeting organised? | | | | | | | - once a week - once a month - other, please specify frequency: | |
| **28** | Does your facility systematically assess the experiences of relatives of residents regarding provided care? | | | | | | | - yes, for all residents - yes, for most residents - yes, for some residents - no, never | |
| **29** | Are specific guidelines, such as an end-of-life care pathway, used for the last 3 days of life of a resident in need of palliative care in your facility? | | | | | | | - yes - no | |
|  | a. If yes, which guidelines are used? | please specify: | | | | | | | |

**Thank you very much for taking time to complete this questionnaire**
