# Supplementary material for: Technical-efficiency analysis of end-of-life care in long-term care facilities within Europe: A cross-sectional study of deceased residents in 6 EU countries (PACE)
Source: PLoS One. 2018 Sep 25;13(9):e0204120. doi: 10.1371/journal.pone.0204120 (PMC6155520; doi:10.1371/journal.pone.0204120)
Supplement: S1 File — (ZIP) [file pone.0204120.s002.zip › PACE_questionnaire_LTCF_staff_resident_study1.doc]

**LONG TERM CARE FACILITY – STAFF**

**Questionnaire to be completed by long term care facility staff regarding the deceased resident**

We would like to ask you to fill in this questionnaire regarding the care provided for the resident mentioned on the enclosed form. As an important care provider, your experiences are extremely valuable and your contribution to this research is indispensable. Filling in this questionnaire will take about 30 minutes of your time.

*Please provide a single answer to each question, unless otherwise indicated. In some cases you can skip questions. This will be indicated as “🡪 skip to question …”. All answers are treated strictly confidentially and processed anonymously. Please send the completed questionnaire directly to the researchers, using the enclosed envelope (no stamp needed).*

**Thank you very much for your willingness to spend time to complete this questionnaire.**

Date of completion of this questionnaire

|  |  |  |  |  |  | 2 | 0 | 1 | 0 |
| --- | --- | --- | --- | --- | --- | --- | --- | --- | --- |

daY	mONTH	YEAR

Health status and treatments during last month of life

(In case the resident stayed in the long term care facility for less than one month please fill in from time of admission to the long term care facility)

| **1** | During the last month of life, did the resident suffer from one or more of the following diseases or events?  *(multiple answers possible)* | - pneumonia |
| --- | --- | --- |
|  |  | - febrile episode (other than caused by pneumonia) |
|  |  | - eating or drinking problem |
|  |  | - hip fracture |
|  |  | - stroke |
|  |  | - gastrointestinal bleeding |
|  |  | - cancer |
|  |  | - other important disease or event, please specify: |

| **2** | Which treatments did the resident receive during the following periods in the last month of life?  *(please indicate all received treatments regardless of their location e.g. hospital or long term care facility)* | | | | | | | | | |
| --- | --- | --- | --- | --- | --- | --- | --- | --- | --- | --- |
|  | *(Multiple answers possible. For each line, please tick at least one box)* | Last month, excluding last week of life | | | | Last week of life | | | Not received | |
|  | Enteral administration of nutrition (tube feeding, e.g. nasogastric tube or PEG) |  | | | |  | | |  | |
|  | Parenteral administration of nutrition (intravenous administration of nutrients) | |  | | |  | | |  | |
|  | Artificial (non-oral) fluid administration: intravenous therapy and/or subcutaneous rehydration | |  | | |  | | |  | |
|  | Resuscitation (CPR or Cardio Pulmonary Resuscitation) | |  | | |  | | |  | |
|  | Artificial ventilation | |  | | |  | | |  | |
|  | Blood transfusion | |  | | |  | | |  | |
|  | Chemo/radiotherapy | |  | | |  | | |  | |
|  | Dialysis (renal replacement therapy) | |  | | |  | | |  | |
|  | Surgery, please specify type of surgery: | |  | | |  | | |  | |
|  | Antibiotics (any type) | |  | | |  | | |  | |
|  | Statins | |  | | |  | | |  | |
|  | Antidiabetics | |  | | |  | | |  | |
|  | New oral anticoagulantia (Xarelto, Pradaxa, …) | |  | | |  | | |  | |
| **3** | Please think back over the last month of life of the resident. Here are some statements that have been considered important during the last phase of life. How true is each statement for (the situation of) the resident? *(for each line, please tick a single box in the column that applies)* | | | | | | | | | |
|  |  | | | **not**  **at all** | a little | | a moderate amount | quite a bit | | **completely** |
| **A** | There was a nurse or aide with whom the resident felt comfortable | | |  |  | |  |  | |  |
| **B** | The resident received affectionate touch daily | | |  |  | |  |  | |  |
| **C** | The resident appeared to be at peace | | |  |  | |  |  | |  |
| **D** | The resident’s physician knew him or her as a whole person including life and personality | | |  |  | |  |  | |  |
| **E** | The resident had treatment preferences in writing | | |  |  | |  |  | |  |
| **F** | The resident indicated he or she was prepared to die | | |  |  | |  |  | |  |
| **G** | The resident’s funeral was planned | | |  |  | |  |  | |  |
| **H** | The resident had named a decision-maker in the event that he or she was no longer able to make decisions | | |  |  | |  |  | |  |
| **I** | The resident maintained his or her sense of humour | | |  |  | |  |  | |  |
| **J** | The resident’s dignity was maintained | | |  |  | |  |  | |  |
| **K** | The resident’s clothes and body were clean | | |  |  | |  |  | |  |

Health status 1 month before death

| **4** | | Cognitive performance scale (**CPS**). | | |
| --- | --- | --- | --- | --- |
|  | | a. Comatose 1 month before death | - yes 🡪 **continue to question 5** | |
|  | |  | - no | |
|  | | b. Short-Term Memory 1 month before death   *(seemed or appeared to recall after 5 minutes)* | - memory OK - memory problem | |
|  | | c. Cognitive skills for daily decision making   1 month before death  *(ability to make everyday decisions, e.g. when to get out of bed, which clothes to wear)* | - independent – *decisions were consistent and reasonable* | |
|  | |  | - modified independent – *only some difficulty in new situations* | |
|  | |  | - moderately impaired – *poor decisions, required supervision* | |
|  | |  | - severely impaired – *never or rarely made decisions* | |
|  | | d. Understood by others   1 month before death  *(ability to express or communicate information – verbal and non-verbal)* | - understood – *expressed ideas clearly* | |
|  | |  | - usually understood – *difficulty finding words of finishing thoughts* | |
|  | |  | - sometimes understood – *ability limited to concrete requests* | |
|  | |  | - rarely/never understood | |
|  | | e. Self-performance in eating   1 month before death  *(how resident ate and drank, regardless of skill, including different ways of feeding including e.g. tube feeding)* | - independent | |
|  | |  | - supervision | |
|  | |  | - limited assistance | |
|  | |  | - extensive assistance | |
|  | |  | - total dependence | |
|  | |  | - not applicable | |
| **5** | Bedford Alzheimer Nursing Severity-Scale (BANS-S) Please choose one item for each of the following 7 functions, indicating the condition of the resident 1 month before death. | | | |
|  |  | | | |
|  | a. Dressing | | | **e.** Mobility |
|  | - Usually is independent | | | - Always able to walk independently |
|  | - Requires minimal assistance | | | - Sometimes able to walk independently |
|  | - Requires moderate assistance but is not totally dependent | | | - Able to walk only with help |
|  | - Totally dependent | | | - Unable to walk even with help |
|  |  | | |  |
|  | **b.** Sleeping | | | **f.** Muscles |
|  | - Usually has a regular sleep-wake cycle | | | - Very flexible and has full joint motion |
|  | - Sometimes has an irregular sleep-wake cycle | | | - Somewhat flexible with some joint motion impairment |
|  | - Frequently exhibits irregular sleep-wake cycle | | | - Somewhat rigid |
|  | - Severely disrupted sleep-wake cycle | | | - Contracted |
|  |  | | |  |
|  | **c.** Speech | | | **g.** Eye contact |
|  | - Completely intact ability to speak | | | - Eye contact is maintained |
|  | - Somewhat decreased ability to speak | | | - Eye contact is usually maintained |
|  | - Moderately decreased ability to speak | | | - Eye contact is rarely maintained |
|  | - Totally mute | | | - Never maintains eye contact |
|  |  | | |  |
|  | **d.** Eating | | |  |
|  | - Eats independently | | |  |
|  | - Requires minimal assistance and/or coaxing | | |  |
|  | - Requires moderate assistance and/or coaxing | | |  |
|  | - Completely dependent | | |  |
| **6** | | Did the resident fit all 3 criteria of the following description 1 month before death? *(*Global Deterioration Scale *stage 7)* | | |
|  | | * A**ll verbal abilities are lost over the course of this stage**.  Early in this stage words and phrases are spoken but speech is very circumscribed. Later there is no serviceable speech at all - only unintelligible utterances with rare emergence of seemingly forgotten words and phrases.  *** Incontinent; requires assistance toileting and feeding**.  *** Basic psychomotor skills** (e.g. ability to walk) **are lost with the progression of this stage**. The brain appears to no longer be able to tell the body what to do. Generalized rigidity and developmental neurologic reflexes are frequently present. | | |
|  | | - yes - no | | |

Dementia

| **7** | In your opinion, did the resident have dementia? | - yes |
| --- | --- | --- |
|  |  | - no 🡪 **continue to question 8** |
|  | a. How long did the resident suffer from dementia? (enter the number of months or years) | months OR years |
|  | b. Did a doctor ever diagnose the resident with dementia? | - yes |
|  |  | - no |
|  | c. What stage of dementia do you think the   resident had at the time of admission? | - early stage (mild dementia) - intermediary stage (moderate dementia) - later stage (severe dementia) - none of the above |
|  | d. What stage of dementia do you think the   resident had at the time of death? | - early stage (mild dementia) - intermediary stage (moderate dementia) - later stage (severe dementia) |

Health status during last week of life

| **8** | Did the resident have decubitus (pressure ulcers) during the last week of life? | - yes |
| --- | --- | --- |
|  |  | - no **🡪 continue to question 9** |
|  | a. Did the resident have superficial or deep ulcers? Superficial or deep ulcers are defined as: Full thickness skin loss involving damage necrosis of subcutaneous tissue that may extend down to underlying fascia or more extensive destruction. | yes  no |
|  |  |  |
|  |  |  |
|  |  |  |
| **9** | Were trunk or limb restraints used during the last week of life? | - used daily - used less frequently than daily - not used |
| **10** | EuroQoL: Please indicate which statements best describe the health of the resident during the last week of life. | |
|  | **a.** Mobility | d. Pain/discomfort |
|  | - no problems in walking about | - no pain or discomfort |
|  | - slight problems in walking about | - slight pain or discomfort |
|  | - moderate problems in walking about | - moderate pain or discomfort |
|  | - severe problems in walking about | - severe pain or discomfort |
|  | - confined to bed | - extreme pain or discomfort |
|  | **b.** Self-Care | e. Anxiety/depression |
|  | - no problems washing or dressing oneself | - not anxious or depressed |
|  | - slight problems washing or dressing oneself | - slightly anxious or depressed |
|  | - moderate problems washing or dressing oneself | - moderately anxious or depressed |
|  | - severe problems washing or dressing oneself | - severely anxious or depressed |
|  | - unable to wash or dress oneself | - extremely anxious or depressed |
|  | c. Usual activities (e.g. housework, family or leisure activities) |  |
|  | - no problems doing ones usual activities |  |
|  | - slight problems doing ones usual activities |  |
|  | - moderate problems doing ones usual activities |  |
|  | - severe problems doing ones usual activities |  |
|  | - unable to do usual activities |  |

**11**

- We would like to know how good or bad the health of the resident was during the last week of life.
- This scale is numbered from 0 to 100.
- 100 means the best health you can imagine.
  0 means the worst health you can imagine.
- Mark an X on the scale to indicate how the health of the resident was during the last week of life.
- Now, please write the number you marked on the scale in the box below.

The health of the resident

during the last week of life =

10

0

20

30

40

50

60

80

70

90

100

5

15

25

35

45

55

75

65

85

95

The best health

you can imagine

The worst health

you can imagine

Dying process

| **12** | EOLD-CAD: When you think about the resident's physical and emotional state, can you please indicate to what extent he or she experienced the following during the last week of life? (*for each line, please tick a single box in the column that applies*) \| a lot \| somewhat \| not at all; a. discomfort \| \| \|; b. pain \| \| \|; c. restlessness \| \| \|; d. shortness of breath \| \| \|; e. choking \| \| \|; f. gurgling \| \| \|; g. difficulty swallowing \| \| \|; h. fear \| \| \|; i. anxiety \| \| \|; j. crying \| \| \|; k. moaning \| \| \|; l. serenity \| \| \|; m. peace \| \| \|; n. calm \| \| \|; o. resistiveness to care \| \| \| | | | |
| --- | --- | --- | --- | --- |
| **13** | | What was the place of death of the resident? | - long term care facility - hospital - hospice/palliative care unit - other, please specify: |  |
|  | | *In case death was in the hospital, if not in hospital continue to question 14*   a. At whose request did the last admission to the hospital take place?   *(multiple answers possible)* | - the resident - family/relatives of the resident - the GP - nurses/carers at the long term care facility - another person, please specify: |  |
|  | | b. According to you, what were the medical **reasons** for this last admission to the hospital? *(multiple answers possible)* | - a specific diagnosis was required to take further decisions - for specific symptomatic treatment - there was a sudden onset or exacerbation of symptoms or a life-threatening situation - another reason, please specify : |  |
| **14** | Please indicate the manner of dying of the resident in the last 6 hours of life. | | - died quietly/died in his/her sleep |  |
|  |  |  | - was disturbed by/was aware of symptoms *(e.g. breathlessness, pain, restlessness, fear, etc.)* |  |
|  |  |  | - suffered till the end/death was a struggle |  |
|  |  |  | - was found dead |  |
| **15** | Level of consciousness, most frequently occurring in the last week: *(please circle what applies)* | | |  |
|  | Awake and alert / awake / awake but drowsy looking / falling asleep / light sleep / deep looking sleep | | |  |

| **16** | Were any opioids (e.g. Morphine, Oxycodone, Hydromorphone, Fentanyl, Buprenorphine, Tramadol) prescribed in the last 3 days of life? *(if necessary, look up in resident’s file)* | | | | | - yes | |
| --- | --- | --- | --- | --- | --- | --- | --- |
|  |  |  |  |  |  | - no | |
| **17** | Were any antipsychotics (e.g. Haloperidol, Risperidone, Olanzapine, Clotiapine) prescribed in the last 3 days of life? *(if necessary, look up in resident’s file)* | | | | | - yes | |
|  |  |  |  |  |  | - no | |
| **18** | Were any hypnotics and/or sedatives (e.g. Midazolam, Oxazepam, Lorazepam, Lormetazepam, Zopiclone, Zolpidem, Zaleplon) prescribed in the last 3 days of life? *(if necessary, look up in resident’s file)* | | | | | - yes | |
|  |  |  |  |  |  | - no | |
| **19** | Which of the following disorders was the resident suffering from at the time of death?  *(multiple answers possible)* | - malignant cancer, i.e.: | | | | | |
|  |  | - lung | | - bowel | - prostate | | - breast |
|  |  |  | - other malignant cancer, please specify: | | | | |
|  |  | - serious cardiovascular disease (not CVA) | | | | | |
|  |  | - cerebrovascular accident (CVA)/stroke | | | | | |
|  |  | - severe pulmonary disease (e.g. COPD) | | | | | |
|  |  | - severe neurological disease (not dementia) | | | | | |
|  |  | - dementia | | | | | |
|  |  | - severe renal disease | | | | | |
|  |  | - severe diabetes | | | | | |
|  |  | - other serious disease or condition, please specify: | | | | | |
|  |  | - none of the above | | | | | |
| **20** | What was the underlying cause of death as far as you know? | - cancer | | | | | |
|  |  | - cardiovascular disorder (not CVA) | | | | | |
|  |  | - cerebrovascular accident (CVA)/stroke | | | | | |
|  |  | - neurological disease (not dementia) - dementia - respiratory disease | | | | | |
|  |  | - other, please specify: | | | | | |

Resource use in the last month of life

| **21** | During the last month of life, how many nights was the resident cared for in a palliative care unit? | | number of nights   - none | |
| --- | --- | --- | --- | --- |
| **22** | During the last month of life, how many times was the resident admitted to a hospital (for more than 24 hours)? | | number of times   - none **🡪 continue to question 23** | |
|  | a. If the resident was admitted to a hospital during the last month of life, please specify the total number of nights spent in each type of ward | **Type of ward** | | **Number of nights spent in each ward during the last month of life** |
|  |  | Geriatric ward | |  |
|  |  | Psychiatric ward | |  |
|  |  | Internal medicine | |  |
|  |  | Surgery | |  |
|  |  | Neurology | |  |
|  |  | General ward | |  |
|  |  | Intensive care unit | |  |
|  |  | Other, please specify: | |  |
| **23** | During the last month of life, how many times did the resident receive care in a hospital emergency room (for less than 24 hours)? | | | number of times   - none |

| **24** | [Country specific question regarding palliative support. BE: Was the ‘palliative support team’ of the hospital involved in the care for the resident in the hospital?] | | | - yes - no - don’t know |
| --- | --- | --- | --- | --- |
| **25** | | During the last month of life, consider how many times the resident visited or received a visit from a doctor, physiotherapist, psychologist or other health care professional.  *Please specify the number of visits for each type of care received. Please provide your best estimate. If there were no visits, please fill in 0.* | **Type of care** | **Number of visits during last month of life** |
|  |  |  | General practitioner |  |
|  |  |  | Geriatrician |  |
|  |  |  | Neurologist |  |
|  |  |  | Psychiatrist |  |
|  |  |  | Occupational therapist |  |
|  |  |  | Social worker |  |
|  |  |  | Psychologist |  |
|  |  |  | Physiotherapist |  |
|  |  |  | Other (e.g. specialist, please specify) |  |

Care during the last months of life

| **26** | [Country specific question regarding the presence of a palliative care file. BE: Did the resident have a ‘palliatief dossier’?] | - yes - no **🡪 continue to question 27** |
| --- | --- | --- |
|  | a. How long before death was the ‘dossier’ started? | days |
| **27** | According to you, did the resident receive palliative care at any time? | - yes |
|  |  | - no **🡪 continue to question 30** |
|  | a. How long before death was palliative care started? | days *(enter number)* |
| **28** | According to you, did the general practitioner provide palliative care to this resident? | - yes, until death |
|  |  | - yes, not until death |
|  |  | - no |
| **29** | Which of the following persons or organisations were involved in the provision of palliative care for the resident?  *(multiple answers possible)*  [Country-specific answer categories: please include persons and organisations that provide palliative care, relevant to your country] | - coördinerend raadgevend arts (CRA) |
|  |  | - palliatief referentie verpleegkundige van het rusthuis |
|  |  | - palliatieve werkgroep van de instelling - externe palliatieve thuiszorgequipe |
|  |  | - none of the above **🡪 continue to question 30** |
|  | a. Did any of these persons or organisations provide a consultation at the resident’s bedside? *(multiple answers possible)*   [Country-specific answer categories: please include persons and organisations that provide palliative care, relevant to your country] | - ja, de coördinerend raadgevend arts (CRA) - ja, de palliatief referentie verpleegkundige van het rusthuis - ja, de palliatieve thuiszorgequipe - ja, iemand anders, namelijk: - nee |
| **30** | Which psychosocial interventions were given to the resident in the last month of life? | - adjustments of environmental factors (e.g. modified environment for walking around safely) - activity programmes - music therapy - behavioural therapy - experiential approaches (e.g. multi-sensory environment, validation therapy) - other, please specify - none of the above |
| **31** | Which medical or nursing treatments/procedures did the resident receive during the last week of life?  *(multiple responses possible)* | - mouth care |
|  |  | - pain assessment |
|  |  | - prevention of bedsores/pressure ulcers/decubitus (e.g. position changes) |
|  |  | - wound care |
|  |  | - assistance with eating or drinking |
|  |  | - oxygen therapy |
|  |  | - suction for respiratory secretions |
|  |  | - ostomy care (any type urinary ostomy or colostomy) |
|  |  | - other, please specify: |
|  |  |  |
| **32** | Was any spiritual and/or pastoral care provided shortly before death? | - yes (e.g. by a pastoral worker or another spiritual care provider) |
|  |  | - no |

Communication and Advance Care Planning

| **33** | Was the resident capable of expressing his or her wishes at the time of admission? \| | | - yes - partly - no | | | |
| --- | --- | --- | --- | --- | --- | --- |
| **34** | Did the resident ever express specific preferences about a medical treatment he or she did or did not want during the last phase of life? | | - yes | | | |
|  |  |  | - no | | | |
|  |  |  | - I don’t know | | | |
| **35** | Did the resident have a written living will with regard to the following requests?  *(tick all that apply)*  [Country-specific answer categories: add or remove categories if necessary] | | - do not resuscitate in case of a cardiac or respiratory arrest (DNR) | | | |
|  |  |  | - do not transfer to a hospital | | | |
|  |  |  | - discontinue the use of or do not use other treatments, please specify: - request to try all life prolonging measures | | | |
|  |  |  | - none of the above | | | |
| **36** | Did the resident, in a prior living will, give a power to a third party to take decisions for him or her in case he or she would be no longer competent to do so? | | | | - yes | |
|  |  |  |  |  | - no | |
| **37** | Did you ever speak with the resident about medical treatments he or she would or would not want in the last phase of life or about the preferred course of care in the last phase of life? | | | | - yes, only once - yes, several times | |
|  |  |  |  |  | - no | |
| **38** | Did the GP record any of the following agreements in the resident’s records about whether medical actions should or should not be performed in case a problem were to occur and the resident would be unable to decide?  *(multiple answers possible)*  [Country-specific answer categories: add or remove categories if necessary in the same way as in question 35] | | - do not resuscitate in case of a cardiac or respiratory arrest (DNR) | | | |
|  |  |  | - do not transfer to a hospital | | | |
|  |  |  | - discontinue the use of or do not use other treatments, please specify: | | | |
|  |  |  | - request to try all life prolonging measures | | | |
|  |  |  | - none of the above **🡪 continue to question 39** | | | |
|  | a. If one of the above wishes was recorded: Who was involved or consulted when this prior wish was recorded?  *(multiple answers possible)*  [Country-specific terms: here ‘coordinating advisory physician, palliative expert]] | | - the resident | | | |
|  |  |  | - a representative, family member(s) or relative(s) | | | |
|  |  |  | - nurses involved in the care | | | |
|  |  |  | - head nurse | | | |
|  |  |  | - coördinerend raadgevend arts (CRA-arts) (NL: aangepast) | | | |
|  |  |  | - palliatief expert (bv referentievpk, LEIF arts, enz) NL:aangepast | | | |
|  |  |  | - other, please specify: | | | |
| **39** | Did you – prior to a decision – speak with a relative of the resident about medical treatments he or she would or would not want in the last phase of life or about the preferred course of care in the last phase of life? | | | | | - yes |
|  |  |  |  |  |  | - no |
| **40** | Was a contact person mentioned in the resident’s records, which you were able to consult when taking decisions about the end of life in case the resident would be unable to do so? | | | | | - yes |
|  |  |  |  |  |  | - no |
| **41** | Were you always able to contact this resident’s GP when necessary in the last week of life? | | - yes, always, also during office hours and ‘on-call ‘ hours - yes, but only during office hours - no - it was not necessary to contact the GP | | | |
| **42** | To which degree were those involved in care in agreement (consensus) on care and treatment in the last month of the resident’s life? | | | | | |
|  | a. long term care facility staff amongst | b. representative/family amongst themselves | | c. amongst all those involved | | |
|  | themselves | - full consensus | | - full consensus | | |
|  | - full consensus | - consensus on major issues | | - consensus on major issues | | |
|  | - consensus on major issues | - no consensus | | - no consensus | | |
|  | - no consensus | - not involved | |  | | |
|  |  | | | | | |

Your background

| **43** | What is your age? | years |
| --- | --- | --- |
|  |  |  |
| **44** | What is your gender? | - male |
|  |  | - female |
| **45** | How many years have you been working in direct patient care? | years  of which years in long term care facilities: |
| **46** | What is your current position or function?  [Country-specific answer categories: need to be adjusted to each country] | - care assistant |
|  |  | - nurse |
|  |  | - headnurse |
|  |  | - reference nurse palliative care |
|  |  | - other, namely: |
| **47** | For how many residents do you provide direct care on an average working day? | number of residents |
| **48** | What is the highest level of education you completed? | - primary/elementary school or lower |
|  |  | - lower secondary education (basic vocational or intermediate secondary) |
|  |  | - upper/higher secondary education |
|  |  | - tertiary education or higher (higher vocational training or university) |
| **49** | Have you ever had any formal training in palliative care?  *(multiple answers possible)* | - no |
|  |  | - yes, as part of my degree - yes, additional education after my degree - yes, other training (please specify): |
|  |  |  |

If you have any remarks or important information regarding the patient or the care delivered, which has not been covered by the questionnaire, please write this below.

**Thank you very much for taking time to complete this questionnaire**
